# Mutated Ca_V_2.1 channels dysregulate CASK/P2X3 signaling in mouse trigeminal sensory neurons of R192Q Cacna1a knock-in mice

**DOI:** 10.1186/1744-8069-9-62

**Published:** 2013-12-02

**Authors:** Aswini Gnanasekaran, Tanja Bele, Swathi Hullugundi, Manuela Simonetti, Michael D Ferrari, Arn MJM van den Maagdenberg, Andrea Nistri, Elsa Fabbretti

**Affiliations:** 1 Neuroscience Department, International School for Advanced Studies (SISSA), Via Bonomea 265, Trieste 34136, Italy; 2 Department of Neurology, Leiden University Medical Center, Leiden 2300 RC, The Netherlands; 3 Department of Human Genetics, Leiden University Medical Center, Leiden RC 2300, The Netherlands; 4 Center for Biomedical Sciences and Engineering, University of Nova Gorica, Nova Gorica SI-5000, Slovenia; 5 Present address: Pharmacology Institute, University of Heidelberg Germany Heidelberg, Germany

**Keywords:** Pain, ATP, Purinergic signaling, DRG, Trigeminal ganglion, Migraine

## Abstract

**Background:**

ATP-gated P2X3 receptors of sensory ganglion neurons are important transducers of pain as they adapt their expression and function in response to acute and chronic nociceptive signals. The present study investigated the role of calcium/calmodulin-dependent serine protein kinase (CASK) in controlling P2X3 receptor expression and function in trigeminal ganglia from *Cacna1a* R192Q-mutated knock-in (KI) mice, a genetic model for familial hemiplegic migraine type-1.

**Results:**

KI ganglion neurons showed more abundant CASK/P2X3 receptor complex at membrane level, a result that likely originated from gain-of-function effects of R192Q-mutated Ca_V_2.1 channels and downstream enhanced CaMKII activity. The selective Ca_V_2.1 channel blocker ω-Agatoxin IVA and the CaMKII inhibitor KN-93 were sufficient to return CASK/P2X3 co-expression to WT levels. After CASK silencing, P2X3 receptor expression was decreased in both WT and KI ganglia, supporting the role of CASK in P2X3 receptor stabilization. This process was functionally observed as reduced P2X3 receptor currents.

**Conclusions:**

We propose that, in trigeminal sensory neurons, the CASK/P2X3 complex has a dynamic nature depending on intracellular calcium and related signaling, that are enhanced in a transgenic mouse model of genetic hemiplegic migraine.

## Background

P2X3 receptors are predominantly expressed on sensory ganglion neurons where they play an important role in transducing pain signals [1]. A major property of these receptors is the ability to rapidly adapt their function to extracellular milieu changes by trafficking-mediated receptor redistribution, by modulation of receptor function through intracellular kinases, or by interaction with specific scaffold proteins [2-5]. We recently reported that under basal conditions P2X3 receptors are strongly associated with the multifunction scaffold protein calcium/calmodulin-dependent serine protein kinase (CASK) [6]. In the present study we investigated whether the CASK/P2X3 complex was altered and functionally linked to sensitization of P2X3 receptors in transgenic knock-in (KI) mice exhibiting a gain-of-function phenotype of voltage-gated Ca_V_2.1 (P/Q-type) calcium channels, due to a R192Q missense mutation in the channel α1 subunit that causes familial hemiplegic migraine type 1 (FHM-1) [7,8]. Using this KI mouse model, we previously identified multiple Ca_V_2.1 channel interactors (calcineurin, Cdk5 and CaMKII) that modulate P2X3 receptor function in trigeminal sensory neurons [9-12]. In particular, enhanced P2X3 receptor-mediated responses were found in KI neurons that depend on constitutive activation of CaMKII and are reversed by the selective Ca_V_2.1 channel blocker or by the CaMKII inhibitor [9]. Previous studies showed that CASK is associated with calcium channels [13-15] and, thus, provide the rational to explore if the R192Q mutation in KI mice influences CASK/P2X3 assembly and function. The present study aimed at testing, with molecular biology and electrophysiological methods, the properties of the CASK/P2X3 receptor complex in this mouse model expressing gain-of-function of Ca_V_2.1 channels, using primary cultures of trigeminal ganglia that fully retain the basal characteristics of the CASK/P2X3 complex *in vivo*[6].

## Results

### The CASK/P2X3 receptor complex is abundantly expressed in KI ganglia and is modulated by Ca^2+^ influx

**Figure 1:**
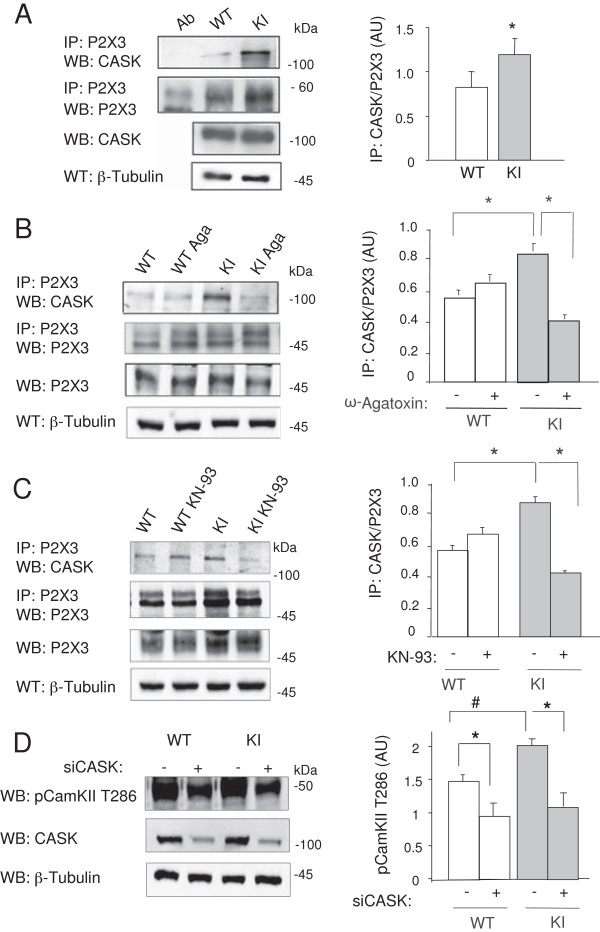
**CASK/P2X3 complex in KI trigeminal neurons. A**, Example of immunopurified P2X3 from trigeminal ganglia probed with anti-CASK antibodies reveals more abundant CASK/P2X3 complex in KI ganglion cultures than in WT ones. Histograms quantify this effect (n = 5, p = 0.038). Ab, indicate signal from unrelated antibody. **B**, CASK/P2X3 co-immunoprecipitation experiments from ganglion cultures demonstrate that CASK/P2X3 complex is sensitive to ω-Agatoxin IVA treatment (400 nM, overnight; Aga). Note the more intense CASK/P2X3 signal in KI samples as seen in **A**. P2X3 immunoprecipitation input and β-Tubulin expression shown as gel loading controls. **C**, CASK/P2X3 complex is sensitive to KN-93 pre-incubation (5 μM, 90 min). **D**, Examples of anti-phospho-CaMKII T286 Western immunoblots of extracts from WT and KI trigeminal cultures in control conditions and after CASK silencing. β-Tubulin is shown as loading control. Histograms quantify the effect (#, p = 0.05; *p = 0.03 for WT and p = 0.025 for KI; n = 3).

In order to study the effects of CASK on P2X3 receptors expressed in WT and KI ganglia, we first compared CASK/P2X3 complex levels in ganglion extracts. Immunoprecipitation experiments showed that the complex was significantly more abundant in KI than in WT samples (Figure 1A; p = 0.038, n = 5). A significant increase (n = 5, p = 0.005) in CASK associated with cell membrane fractions was observed in KI tissue (Additional file 1: Figure S1A), although total CASK lysate preparations did not show any difference between WT or KI samples (Additional file 1: Figure S1B). Further experiments concerning the specificity of the CASK/P2X3 complex, based on immunoprecipitating CASK first and then performing western blotting with P2X3 antibodies, validated our previous findings [6] and are included in Additional file 2: Figure S2A, B.

In analogy to its effect on other receptors (e.g. NMDA receptors; [15]), CASK might exert a role in the process of P2X3 receptor export to surface membranes. In fact, pulled-down biotinylated surface P2X3 receptors showed co-purification with intracellular CASK (Additional file 3: Figure S3), supporting the view that CASK/P2X3 complexes are membrane-bound. In these biotinylation experiments, no difference was observed in the levels of surface membrane CASK in WT and KI samples (Additional file 3: Figure S3).

We further explored whether the origin of the stronger CASK/P2X3 association in KI samples could be directly linked to the Ca_V_2.1 R192Q gain-of-function and concomitant higher CaMKII activation [9]. To this end, we used selective pharmacological tools to specifically block either Ca_V_2.1 channel function or apply inhibitors of downstream CaMKII activity. Trigeminal ganglion cultures were treated with the selective Ca_V_2.1 channel blocker ω-Agatoxin IVA (400 nM, 15 h) and then tested for CASK/P2X3 protein interaction by immunoprecipitation. Immunoprecipitation experiments with purified P2X3 receptors and CASK expression (Figure 1B) confirmed that CASK/P2X3 receptor interaction was stronger in KI cultures and that it was significantly reversed by ω-Agatoxin IVA application. Interestingly, CASK/P2X3 complex in WT culture was not affected by the channel blocker suggesting that larger CASK/P2X3 interaction in KI neurons was likely due to the enhanced Ca_V_2.1 channel activity.

Likewise, after cultures treatment with the CaMKII inhibitor KN-93 (5 μM, 90 min, Figure 1C), the CASK/P2X3 interaction was significantly inhibited in KI, but not in WT (Figure 1C). In addition, after RNA silencing of CASK expression [6], there was a significant decrease in CaMKII phosphorylation (Figure 1D).

### P2X3 expression and function after siCASK in WT and KI ganglion cultures

**Figure 2:**
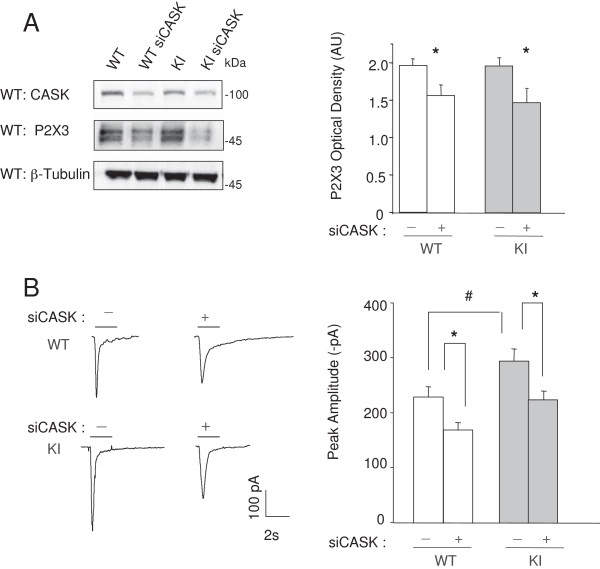
**Effect of siCASK silencing in KI and WT neurons. A**, Example of Western immunoblotting for CASK, P2X3 and β-tubulin, in control and after siCASK silencing. Note the significant decrease in P2X3 receptor expression in both WT and KI samples. Histograms show significant reduction of P2X3 protein expression in silenced samples (*p = 0.026 for WT and p = 0.04 for KI; n = 5, number of experiments). **B**, representative examples of currents induced by α,β-meATP (α,β, black bar, 10 μM) from WT or KI trigeminal neurons in control or after siCASK. Histograms show quantification of mean current amplitude values (WT: 230 ± 19 pA or 170 ± 13 pA; KI: 296 ± 92 pA or 225 ± 16 pA for control or siCASK respectively). # p = 0.036, *p = 0.02 for WT and p = 0.019 for KI; n = 15–23 cells.

Our recent findings [6] that showed how siCASK significantly lowered P2X3 expression in trigeminal ganglion cultures, have been further validated in the present study in which no difference between WT and KI cultures was observed as a consequence of siCASK (Figure 2A). To further explore functional consequence of CASK/P2X3 complex in the KI model, patch clamp experiments were carried out (Figure 2B). Sample P2X3 receptor currents elicited by pulse application of the selective agonist α,β-methylene-adenosine-5′-triphosphate (α,β-meATP; 10 μM) were clearly smaller after siCASK silencing, but proportionally similar in WT and KI neurons. As expected [9], KI neuronal currents were constitutively larger than WT ones (Figure 2B).

## Discussion

The principal finding of the present study is the demonstration that the R192Q missense mutation in the α1 subunit of Ca_V_2.1 calcium channels, that confers gain of function to these channels, is associated with more abundant CASK/P2X3 complex in trigeminal sensory neurons with a mechanism clearly dependent on Ca_V_2.1 channel function and CaMKII activation. This result outlines a molecular mechanism whereby P2X3 receptors are retained and display stronger activity in KI neurons and can, therefore, contribute to sensitization to painful stimuli.

Our former studies have shown that P2X3 receptors of trigeminal sensory neurons are under the influence of endogenous kinases such as Csk and Cdk5, which regulate the basal operational activity of these receptors [9,16]. We have recently observed a new member of this class of regulators, namely CASK, which strongly controls P2X3 receptor expression and function at the plasma membrane of mouse trigeminal neurons under physiological conditions [6,17]. The present report shows that CASK is more associated with P2X3 receptors in KI ganglia and in culture, and that this association is largely dependent on Ca_V_2.1 calcium channel and CAMKII activity as their specific pharmacological blockers reversed the effect. When CASK expression was inhibited by siRNA silencing, the P2X3 receptor expression and function fell to similar levels in WT and KI neurons. In biotinylation experiments, since no difference was observed in CASK fractions from WT or KI surface membrane samples, these experiments are, therefore, consistent with a differential distribution of P2X3 receptors to lipid raft compartments in KI ganglia [3]. When CASK was blocked, the residual P2X3 receptor currents were approximately one-third smaller, providing a first estimate about the extent of the role of CASK in regulating P2X3 receptor activity. Such data suggest that the stronger CASK/P2X3 association in KI cells was an important factor for the observed up-regulation of P2X3 receptors.

**Figure 3:**
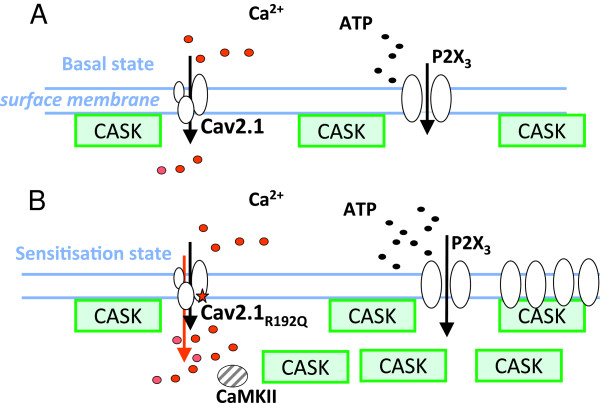
**Proposed mechanism of action of CASK/P2X3 complex in trigeminal sensory neurons.** Schematic representation of CASK, P2X3, and Ca_V_2.1 channel protein interactions at the plasma membrane of trigeminal sensory neurons. **A**, In WT ganglion cells, only a fraction of P2X3 receptors is thought to be associated with CASK that ensures their stable expression. Under these conditions, Ca_V_2.1 channels provide only a minor contribution to Ca^2+^ influx, and the dynamic CASK/P2X3 association ensures physiological responses and P2X3 receptors turn-over [6]. **B**, In KI ganglia, the hyperfunctional Ca_V_2.1 channels result in a stronger Ca^2+^ influx, larger CaMKII activity, and more abundant CASK/P2X3 receptor complexes with improved stability of P2X3 receptors. Sub-membrane vesicle-associated CASK, potentially associated with P2X3 receptor trafficking, is also indicated. Larger release of extracellular ATP [20] facilitates P2X3 responses and may contribute to the process of sensitization of P2X3 receptors.

Figure 3 summarizes an idealized scheme of how we envisage the interaction between certain neuronal proteins affecting P2X3 receptor responsiveness. In WT neurons, only a fraction of P2X3 receptors is thought to be associated with CASK, which ensures their stability at the plasma membrane level [6]. Under these conditions, Ca_V_2.1 channel activity provides only a relatively minor contribution to Ca^2+^ influx [9,18,19] and P2X3 receptors are regulated only to limited extent by CASK (Csk and Cdk5 are not depicted here). The hyper-functional Ca_V_2.1 channels in KI (that require a smaller membrane depolarization to reach the activation threshold [8,20]) induce stronger Ca^2+^ influx and CaMKII activity [9], improving the stability of CASK/P2X3 receptor complexes and their activity at plasma membrane level. Furthermore, these processes probably synergize and add to the already larger release of extracellular ATP by KI ganglia [21], thereby further facilitating P2X3 responses and thus contributing to the process of sensitization of P2X3 receptors [9].

In conclusion, the present data suggest that CASK could play the role of scaffold protein linking different membrane and sub-membrane molecules (including other channels) to elicit further downstream signaling. Since CASK is involved at presynaptic level to modulate synaptic transmitter release [22], it is feasible that this anchoring mechanism may also occur at central level and shows distinct alterations in chronic pain.

## Materials and methods

### Tissue culture

Ca_V_2.1 R192Q KI and WT mice [8] were euthanized by slowly raising levels of CO_2_ in accordance with the Italian Animal Welfare Act and approved by SISSA Ethical Committee. Trigeminal ganglia primary cultures were obtained as previously described [6]. In specific experiments ω-Agatoxin IVA (400 nM; 15 h; Sigma, Milan, Italy) or CaMKII inhibitor KN-93 (5 μM; Sigma) were added to the culture medium for the indicated time [9]. CASK siRNA experiments were performed as previously reported [6].

### Western blotting and immunoprecipitation

Protein extracts and immunoprecipitation experiments and membrane protein biotinylation were performed as previously described [6]. P2X3 receptors were solubilized in 10 mM Tris–HCl (pH 7.5), 150 mM NaCl, 20 mM EDTA, and 1% Triton-X-100 plus protease inhibitors (Roche Products, Welwyn Garden City, UK) as previously reported [3,6]. The non-ionic detergent n-octyl-β-D-glucoside (ODG; [9]) was not used. For western blot quantification, signals expressed in optical density absolute units (AU) were measured with digital imaging system (UVITEC, Cambridge, UK).

### Patch clamp recordings

Currents from siRNA-treated cultures (72 h) were recorded under whole cell voltage clamp mode at holding potential of -65 mV after correcting for the liquid junction potential [6]. P2X3 receptor synthetic agonist α,β-meATP (10 μM; Sigma) was applied (2 s) using a fast superfusion system (RSC-200; BioLogic Science Instruments, Claix, France).

### Data analysis

Data are presented as mean ± standard error. Statistical significance was evaluated with paired Student’s *t*-test or Mann–Whitney rank sum test using OriginPro 7.5 (OriginLab, Northampton, MA, USA).

## Abbreviations

α: β-meATP α,β-methyleneATP; CaMK: Calcium/calmodulin-dependent kinase; CASK: Calcium/calmodulin-dependent serine kinase; TG: Trigeminal ganglia.

## Competing interests

The authors state that the content of this article does not create any competing interest.

## Authors’ contributions

AG carried out biochemical studies and statistical analysis, TB performed further data collection, SH carried out functional studies and statistical analysis, MF, AM provided animal model; EF, MS conceived of the study, EF, AN design and coordination; AG, AM, AN, EF draft the manuscript. All authors read and approved the final manuscript.

## Supplementary Material

Additional file 1: Figure S1 **A**, **B** Example of a Western blot experiment of total trigeminal ganglia extracts **(A)** or total membrane or ganglia samples **(B)** from WT and KI mice tested with anti-CASK antibodies. Histogram quantifications show no difference of total CASK expression in WT and KI (n = 3; p > 0.05), and a significant enrichment of total membrane-associated CASK expression in KI vs WT (n = 5, p = 0.005). Actin was used as gel loading control. Click here for file

Additional file 2: Figure S2 **A**, Western blot experiment of trigeminal ganglia extracts after immunoprecipitation with anti-CASK and revealed with anti-CASK or anti-P2X3 antibodies. β-Actin signals quantify immunoprecipitation input. **B**, Membrane protein biotinylation experiments of trigeminal ganglia cultures in control and after siP2X3, analysed with western blot and probed with anti-CASK or anti-P2X3 antibodies, as indicated. Signals from total extracts (left) and streptavidin pull-down (right) of biotinylated samples are shown. No difference in membrane-bound CASK after siP2X3 is found β-Actin is used as gel loading control. Note lack of β-Actin in pull-down samples. Click here for file

Additional file 3: Figure S3 Membrane protein biotinylation experiments of WT and KI trigeminal ganglia cultures in control and after siP2X3, revealed with western blot and probed with anti-CASK and anti-P2X3 antibodies. Signals from total extracts (left) and streptavidin pull-down (right) of biotinylated samples are shown. β-Actin is used as gel loading control. Histograms quantify larger surface P2X3 receptors in trigeminal ganglia cultures from KI mice and no changes in surface-associated CASK between WT and KI samples (n = 3, *p < 0.05). Click here for file

## References

[B1] Burnstock G Purinergic signalling and disorders of the central nervous system Nat Rev Drug Discov 2008 7 575 590 10.1038/nrd2605 18591979

[B2] Chen XQ Wang B Wu C Pan J Yuan B Su YY Jiang XY Zhang X Bao L Endosome-mediated retrograde axonal transport of P2X3 receptor signals in primary sensory neurons Cell Res 2012 22 677 696 10.1038/cr.2011.197 22157653 PMC3317558

[B3] Gnanasekaran A Sundukova M van den Maagdenberg AM Fabbretti E Nistri A Lipid rafts control P2X3 receptor distribution and function in trigeminal sensory neurons of a transgenic migraine mouse model Mol Pain 2011 7 77 10.1186/1744-8069-7-77 21958474 PMC3193817

[B4] Vacca F D’Ambrosi N Nestola V Amadio S Giustizieri M Cucchiaroni ML Tozzi A Velluz MC Mercuri NB Volonté C N-Glycans mutations rule oligomeric assembly and functional expression of P2X3 receptor for extracellular ATP Glycobiology 2011 21 634 643 10.1093/glycob/cwq211 21186285

[B5] Vacca F Giustizieri M Ciotti MT Mercuri NB Volonté C Rapid constitutive and ligand-activated endocytic trafficking of P2X receptor J Neurochem 2009 109 1031 1041 10.1111/j.1471-4159.2009.06029.x 19519775

[B6] Gnanasekaran A Sundukova M Hullugundi S Birsa N Bianchini G Hsueh YP Nistri A Fabbretti E CASK is a new intracellular modulator of P2X3 receptors J Neurochem 2013 126 102 112 10.1111/jnc.12272 23600800

[B7] Ophoff RA Terwindt GM Vergouwe MN van Eijk R Oefner PJ Hoffman SM Lamerdin JE Mohrenweiser HW Bulman DE Ferrari M Haan J Lindhout D van Ommen GJ Hofker MH Ferrari MD Frants RR Familial hemiplegic migraine and episodic ataxia type-2 are caused by mutations in the Ca^2+^ channel gene CACNL1A4 Cell 1996 87 543 552 10.1016/S0092-8674(00)81373-2 8898206

[B8] van den Maagdenberg AM Pietrobon D Pizzorusso T Kaja S Broos LA Cesetti T van de Ven RC Tottene A van der Kaa J Plomp JJ Frants RR Ferrari MD A Cacna1a knockin migraine mouse model with increased susceptibility to cortical spreading depression Neuron 2004 41 701 710 10.1016/S0896-6273(04)00085-6 15003170

[B9] Nair A Simonetti M Birsa N Ferrari MD van den Maagdenberg AM Giniatullin R Nistri A Fabbretti E Familial hemiplegic migraine Ca_v_2.1 channel mutation R192Q enhances ATP-gated P2X3 receptor activity of mouse sensory ganglion neurons mediating trigeminal pain Mol Pain 2010 6 48 10.1186/1744-8069-6-48 20735819 PMC2940876

[B10] Nair A Simonetti M Fabbretti E Nistri A The Cdk5 kinase downregulates ATP-gated ionotropic P2X3 receptor function via serine phosphorylation Cell Mol Neurobiol 2010 30 505 509 10.1007/s10571-009-9483-2 19960242 PMC11498751

[B11] Simonetti M Giniatullin R Fabbretti E Mechanisms mediating the enhanced gene transcription of P2X3 receptor by calcitonin gene-related peptide in trigeminal sensory neurons J Biol Chem 2008 283 18743 18752 10.1074/jbc.M800296200 18460469

[B12] Fabbretti E Nistri A Regulation of P2X3 receptor structure and function CNS Neurol Disord Drug Targets 2012 11 687 698 10.2174/187152712803581029 22963434

[B13] Maximov A Südhof TC Bezprozvanny I Association of neuronal calcium channels with modular adaptor proteins J Biol Chem 1999 274 24453 24456 10.1074/jbc.274.35.24453 10455105

[B14] Spafford JD Zamponi GW Functional interactions between presynaptic calcium channels and the neurotransmitter release machinery Curr Opin Neurobiol 2003 13 308 314 10.1016/S0959-4388(03)00061-8 12850215

[B15] Jeyifous O Waites CL Specht CG Fujisawa S Schubert M Lin EI Marshall J Aoki C de Silva T Montgomery JM Garner CC Green WN SAP97 and CASK mediate sorting of NMDA receptors through a previously unknown secretory pathway Nat Neurosci 2009 12 1011 1019 10.1038/nn.2362 19620977 PMC2779056

[B16] D’Arco M Giniatullin R Leone V Carloni P Birsa N Nair A Nistri A Fabbretti E The C-terminal Src inhibitory kinase (Csk)-mediated tyrosine phosphorylation is a novel molecular mechanism to limit P2X3 receptor function in mouse sensory neurons J Biol Chem 2009 284 21393 21401 10.1074/jbc.M109.023051 19509283 PMC2755864

[B17] Volonté C Burnstock G P2X3 receptor: a novel‘CASKade’ of signaling? J Neurochem 2013 126 1 3 23682654 10.1111/jnc.12282

[B18] Ikeda M Matsumoto S Classification of voltage-dependent Ca^2+^ channels in trigeminal ganglion neurons from neonatal rats Life Sci 2003 73 1175 1187 10.1016/S0024-3205(03)00414-4 12818725

[B19] Tao J Liu P Xiao Z Zhao H Gerber BR Cao YQ Effects of familial hemiplegic migraine type 1 mutation T666M on voltage-gated calcium channel activities in trigeminal ganglion neurons J Neurophysiol 2012 107 1666 1680 10.1152/jn.00551.2011 22190617 PMC3311679

[B20] Tottene A Conti R Fabbro A Vecchia D Shapovalova M Santello M van den Maagdenberg AM Ferrari MD Pietrobon D Enhanced excitatory transmission at cortical synapses as the basis for facilitated spreading depression in Ca_v_2.1 knockin migraine mice Neuron 2009 61 762 773 10.1016/j.neuron.2009.01.027 19285472

[B21] Franceschini A Nair A Bele T van den Maagdenberg AM Nistri A Fabbretti E Functional crosstalk in culture between macrophages and trigeminal sensory neurons of a mouse genetic model of migraine BMC Neurosci 2012 13 143 10.1186/1471-2202-13-143 23171280 PMC3511260

[B22] Hsueh YP The role of the MAGUK protein CASK in neural development and synaptic function Curr Med Chem 2006 13 1915 1927 10.2174/092986706777585040 16842202

