# Supplementary figures and images for: Mutated CaV2.1 channels dysregulate CASK/P2X3 signaling in mouse trigeminal sensory neurons of R192Q Cacna1a knock-in mice
Source: Mol Pain. 2013 Dec 2;9:62. doi: 10.1186/1744-8069-9-62 (PMC4220808; doi:10.1186/1744-8069-9-62)

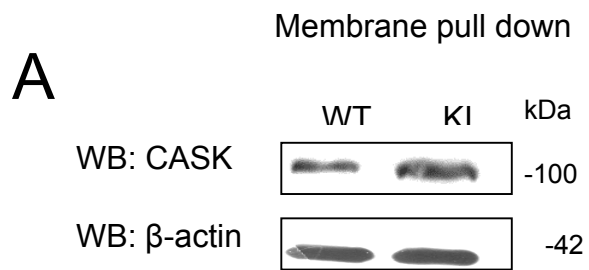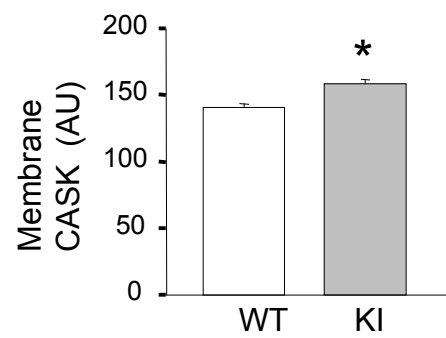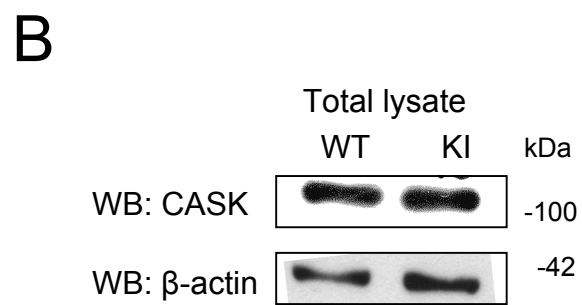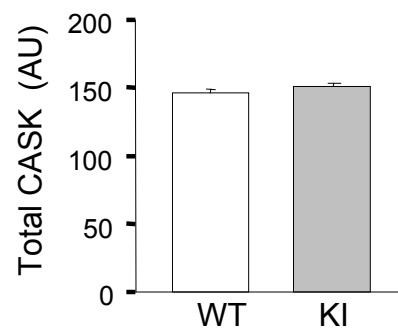

Supplement: Additional file 1: Figure S1 — A, B Example of a Western blot experiment of total trigeminal ganglia extracts (A) or total membrane or ganglia samples (B) from WT and KI mice tested with anti-CASK antibodies. Histogram quantifications show no difference of total CASK expression in WT and KI (n = 3; p > 0.05), and a significant enrichment of total membrane-associated CASK expression in KI vs WT (n = 5, p = 0.005). Actin was used as gel loading control. [file 1744-8069-9-62-S1.pdf]

Suppl Fig. 2

A

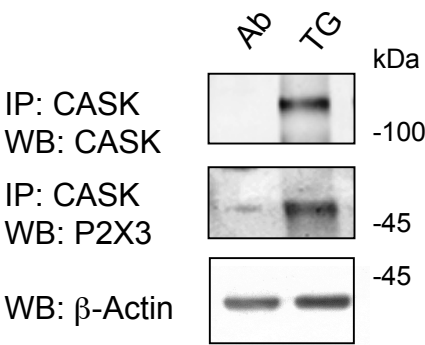

B

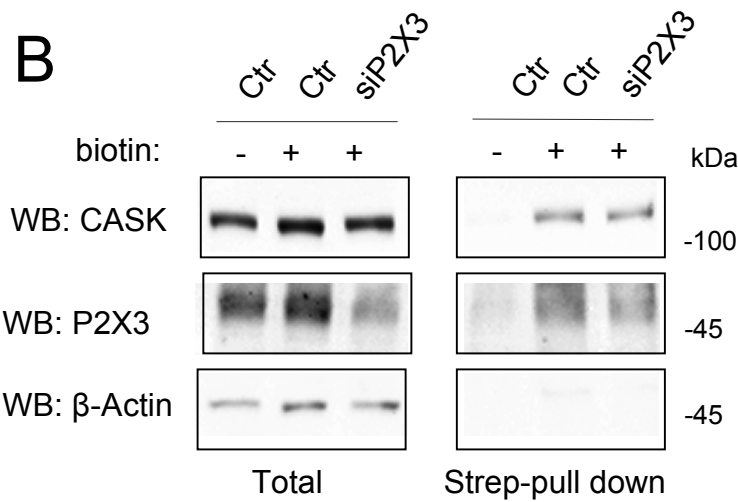

Supplement: Additional file 2: Figure S2 — A, Western blot experiment of trigeminal ganglia extracts after immunoprecipitation with anti-CASK and revealed with anti-CASK or anti-P2X3 antibodies. β-Actin signals quantify immunoprecipitation input. B, Membrane protein biotinylation experiments of trigeminal ganglia cultures in control and after siP2X3, analysed with western blot and probed with anti-CASK or anti-P2X3 antibodies, as indicated. Signals from total extracts (left) and streptavidin pull-down (right) of biotinylated samples are shown. No difference in membrane-bound CASK after siP2X3 is found β-Actin is used as gel loading control. Note lack of β-Actin in pull-down samples. [file 1744-8069-9-62-S2.pdf]

# Suppl Fig. 3

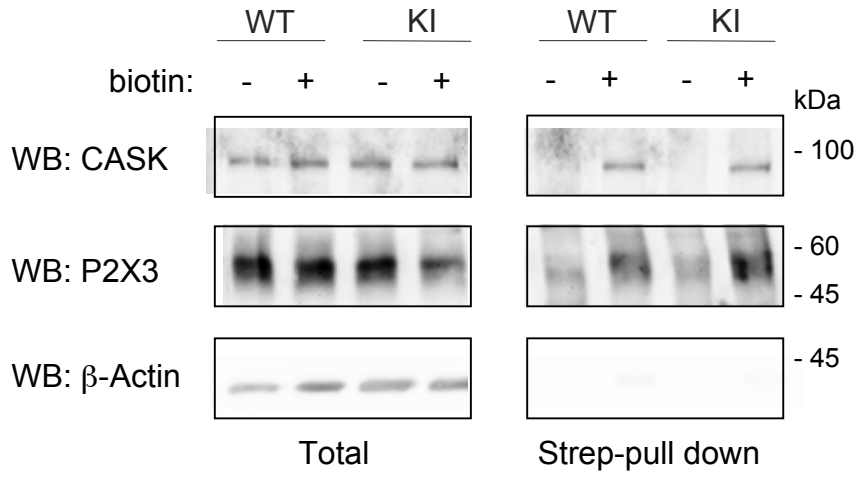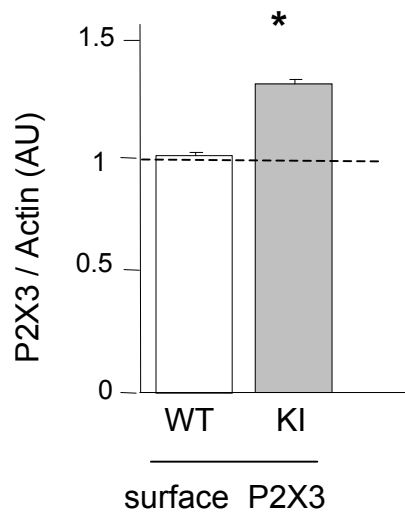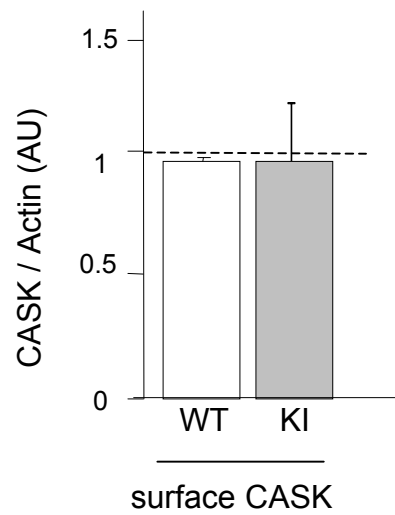

Supplement: Additional file 3: Figure S3 — Membrane protein biotinylation experiments of WT and KI trigeminal ganglia cultures in control and after siP2X3, revealed with western blot and probed with anti-CASK and anti-P2X3 antibodies. Signals from total extracts (left) and streptavidin pull-down (right) of biotinylated samples are shown. β-Actin is used as gel loading control. Histograms quantify larger surface P2X3 receptors in trigeminal ganglia cultures from KI mice and no changes in surface-associated CASK between WT and KI samples (n = 3, *p < 0.05). [file 1744-8069-9-62-S3.pdf]
